# Do communication training programs improve students’ communication skills? - a follow-up study

**DOI:** 10.1186/1756-0500-5-486

**Published:** 2012-09-05

**Authors:** Anne Simmenroth-Nayda, Cora Weiss, Thomas Fischer, Wolfgang Himmel

**Affiliations:** 1 Department of General Practice / Family Medicine University of Göttingen, Humboldtalle 38, 37077, Göttingen, Germany

## Abstract

**Background:**

Although it is taken for granted that history-taking and communication skills are learnable, this learning process should be confirmed by rigorous studies, such as randomized pre- and post-comparisons. The purpose of this paper is to analyse whether a communication course measurably improves the communicative competence of third-year medical students at a German medical school and whether technical or emotional aspects of communication changed differently.

**Method:**

A sample of 32 randomly selected students performed an interview with a simulated patient before the communication course (pre-intervention) and a second interview after the course (post-intervention), using the Calgary-Cambridge Observation Guide (CCOG) to assess history taking ability.

**Results:**

On average, the students improved in all of the 28 items of the CCOG. The 6 more technically-orientated communication items improved on average from 3.4 for the first interview to 2.6 in the second interview (p < 0.0001), the 6 emotional items from 2.7 to 2.3 (p = 0.023). The overall score for women improved from 3.2 to 2.5 (p = 0.0019); male students improved from 3.0 to 2.7 (n.s.). The mean interview time significantly increased from the first to the second interview, but the increase in the interview duration and the change of the overall score for the students’ communication skills were not correlated (Pearson’s r = 0.03; n.s.).

**Conclusions:**

Our communication course measurably improved communication skills, especially for female students. These improvements did not depend predominantly on an extension of the interview time. Obviously, “technical” aspects of communication can be taught better than “emotional” communication skills.

## Background

It is widely accepted that physicians require good history taking and communication skills [1]. They are particularly important in primary care settings where diagnoses often may be obtained by an attentive history taking alone. Moreover, patient outcomes such as drug adherence, patient satisfaction and coping with illness depend, amongst others, on the doctor’s communication abilities [1-7]. As history taking and communication with patients are frequent and essential tasks, these skills should be taught early and repeatedly throughout medical education with the support of simulated patients (SP), combined with structured feedback. They should be taught more in a problem-based method (“experimental”) than with instructional teaching methods [6-12].

Although it is taken for granted that history taking and communication skills are learnable [12,13], there is a paucity of rigorous studies that have directly measured and demonstrated a learning progress on basis of a proper design that allows for valid conclusion [14,15]. Many studies are based on paper-pencil questionnaires which assess only the students’ knowledge about communications skills; other authors have relied on self-assessment methods [16,17], although students tend to either overestimate or underestimate their own skills [18]. In their review, Aspegren et al. [8] examined the evidence for the impact of communication skills training in medical students and found a lack of studies from other than English-speaking countries, e.g. Germany.

There is at least one study, conducted at the John Hopkins University School of Medicine that used a randomized design with a control group to measure the effectiveness of a communication course on the students’ ability for clinical reasoning [19]. Interestingly, while the clinical reasoning skills differed markedly between the control and intervention group, the students’ communication skills did not differ, or only to a lesser degree, after the intervention. Moreover, since the students’ ability was not measured before and after intervention by tutors or SP, it is difficult to decide whether communication skills improved and if so, which skills did or did not improve.

In Germany, interactive teaching methods have rapidly developed in recent years and half of all medical faculties are working with SP or using OSCEs [7]. In 2004, a new law (“Approbationsordnung für Ärzte”) regarding the education of medical doctors came into effect which supports further innovative teaching initiatives and the acquisition of communicative competence. Some medical schools have a systematic curriculum for teaching communication skills [7,20,21] and a consensus paper has been published by the German Society of Medical Education in 2008, which contained a guideline about which social and communicative competencies medical students should have achieved by the end of their medical studies [22].

At the Göttingen Medical school a new compulsory list of learning goals was established. For the first time, communicative skills like “the student is able to take a complete medical history” were mentioned. Notwithstanding these positive signals, we felt that our medical colleagues from other disciplines had still severe doubts as to the usefulness of teaching communicative competencies and the success of our courses. Therefore, we saw the necessity of assessing our new teaching methods.

In a randomized pre-post-design, we wanted to explore whether a communication course for third-year medical students has a measureable effect on communication skills. While this study primarily responds to the new situation at our faculty, including the need to assess our methods, our study question also included aspects that had not been investigated in detail to date. Of particular interest was whether certain communication skills improved more than others and if male and female students benefited similarly.

## Method

### Context

At the Göttingen Medical school, all third-year students visit a “basic clinical skills course” including manual (e.g. injections, ECG, wound-suturing) and communication skills (e.g. history taking and basic variables of communication techniques such as empathy, active listening, nonverbal communication). This is the first course with systematic teaching of communication skills after two years of basic scientific skills (“Vorklinik”). Ten modules, each lasting 3 full hours are taught over 12 weeks. With small variations, this course has taken place since 2004.

Components of the basic clinical skills course included, besides others

· type of questions

· body language

· techniques like paraphrasing and reflection of emotions

· complete history taking and

· basics about the patient-physician-relationship.

We are teaching in small groups with 5 to 6 students supervised by a MD or psychologist or rotationally by student tutors, practicing role plays and group-based consultations with simulated patients (SP). SP were trained how to give structured feedback, using the “sandwich-technique” and speaking in “third person” (e.g., “In my role as Ms. Smith, I would prefer if you do not interrupt my first phrase”) [23]. Two other courses are taught parallel: “basics of laboratory diagnostics and pathology”, and “imaging techniques and radiation protection”, both topics without any communicative aspects (only lectures and laboratory rotation).

### Study design and participants

**Figure 1:**
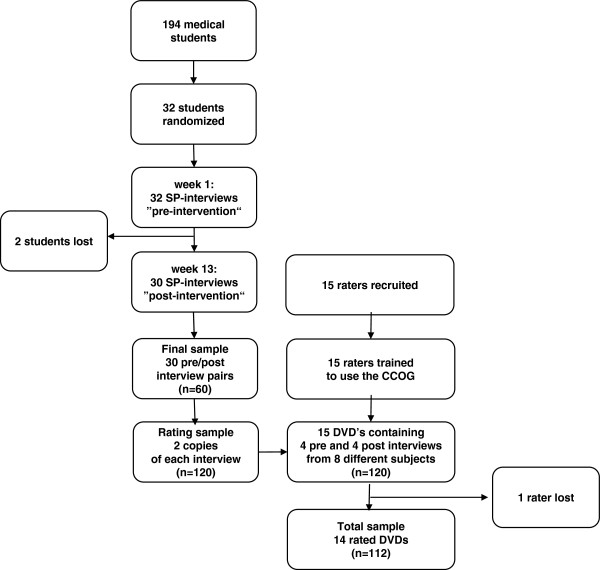
Flowchart to show the sequential steps in this study from selection, randomization and assessment of students (SP: simulated patient).

This was an explorative study. We used a before-and-after design to assess the students' performance before and after an intervention, where the communication course represented the intervention. A gender-stratified randomly selected sample of third-year students taking the basic clinical skills course was drawn by choosing the first male and a female student, according to the alphabetic order, from each of the 8 small sub-groups. They were separated from the rest of the group and did not receive the first lesson of the basic skills course. Instead, they had to perform an SP-interview, not being prepared to do so (Figure 1). Participation was voluntary. The students who agreed to take part gave written informed consent and were informed of the study and that they had to spend an extra lesson at the end of the regular course 3 months later.

Students then performed the initial SP interview, which was recorded. During this interview, only the SP and one of the authors (CW) were present for videotaping. Two experienced SP were trained and portrayed 2 different roles (“allergic rhinitis” and “acute gastritis”). The roles were randomly assigned to the first and second interview. The SPs were instructed to give no feedback and to act as in an exam situation. At the end of the course, the selected students performed a second SP interview with the other SP. All videotapes were converted to digital files and 8 interviews (consisting of 4 first and 4 second interviews) were randomly copied onto different DVDs. Only 2 of the authors (AS and CW) were aware of the status of the interviews.

### Instrument

To assess the quality of the history taking and the ‘doctor-patient’ relationship, we chose the translated and validated short-version (28 items) of the Calgary-Cambridge Observation-Guide (CCOG) [10,12]. This guide is subdivided in 6 sections, which reflect important steps of history taking and consultation:

· initiating the session

· gathering information

· understanding the patient-perspective

· providing structure to the consultation

· building a relationship

· closing the session.

Six items in particular of the CCOG exhibit a more emotional character e.g. “demonstrated respect” or “empathizes with and supports patient” while six other items highlight a more technical aspect of history taking, e.g. “introduces self and role” or “structures logical sequence”. The CCOG contains a 5-point scale (1 = “excellent” and 5 = “deficient”).

### Assessment

A group of voluntarily recruited members of our department (family physicians, psychologists, sociologists) were trained as raters in a 90-minute session. The session comprised a short presentation of the experiment, the CCOG and the rating of an interview. These ratings were discussed with the entire group in detail.

After this instruction, each rater obtained a DVD and had to rate the 8 interviews within the following 8 weeks. Each interview should be assessed by 2 raters. Reminders were sent out by telephone and e-mail. The raters were not engaged in teaching during the semester that this analysis was conducted so as they would not be able to recognize or sympathize with some of the students they would see on the DVD.

### Statistical analysis

All analyses were performed with SAS 9.2. On the basis of the 28 items of the CCOG, we calculated a total score for each student as the unweighted mean of all 28 items and a score for the sub-groups of “technical” and “emotional” items. We compared differences between the first and the second interview for scores on single, technical and emotional items, and the total score, using the Wilcoxon signed rank test or the 2-sample paired t-test, as appropriate.

Correlations between the interview duration and the total score, as well as correlations between the change in the duration of the interview and the change of scores between the first and second interview were determined by Pearson’s coefficient r.

Each student’s interview was assessed by two raters and the scores were the mean of these 2 ratings. Agreement between the two raters was first determined by weighted Kappa. In two instances, only one rater was able to provide an assessment for a student’s SP-interview. Since it is not possible to calculate Kappa with missing values, we chose the “zero” option in SAS and replaced the missing value with the value of the first rater and gave this value a very small weight, close to zero. In a second step, the correlation between the total score for the first interview and the second interview was determined by Pearson’s coefficient r.

### Ethical approval

This study was embedded in the curriculum development at Göttingen Medical School. Neither were patients included nor were any of our interventions invasive; student participation was completely voluntary. Therefore, ethical approval was not deemed necessary.

## Results

### Participants

From 194 third-year students, we selected 16 male and 16 female students; they all agreed to take part in the study and gave informed consent. Table 1 shows the baseline characteristics of the sample. One third of the participants had had a preparatory training (e.g. emergency medical, nursing) before entering medical school.

**Table 1:** Characteristics of participants

**Participants**	**n**	**%**
**Age (years)**		
20 - 24	23	79.3
25 - 29	4	13.7
>30	2	7
**Female**	16	50
**Preparatory Training**		
none	19	63.3
civilian service in medicine*	5	16.6
nursing	4	13.3
paramedic†	2	6.6
**Number of Semesters**		
5 - 6 Semester	24	71
7 - 8 Semester	2	6.7
**9 - 12 Semester**	4	13.3

* In Germany up until 2011, all male adolescents were required to serve 9 months either in the military or in civil service (the latter often in medical or social institutions). We have included here only service in medical institutions.

† While waiting for a university placement or during civilian service, some students are trained as paramedics.

All students performed the first SP-interview; 2 of the 32 students did not perform the second SP-interview; the reason in both cases was “lack of time”. The valid sample of pre/post interview pairs was 30, making a total sample of 60 individual interviews. Since each of these 60 interviews should be rated by two different raters (n=120 ratings), we enrolled 15 raters and supplied each one with interviews from 8 different students (4 first and 4 second interviews). Of these 15 raters, 14 performed the required assessment, resulting in a sample of 112 ratings (Figure 1).

### Inter-rater-reliability

As described above, each interview was rated by two different people. The inter-rater-reliability (weighted kappa) ranged between 0.2 and 0.5 across the different items. We also composed the total scores of each pair of raters and found a correlation of 0.62 (Pearson’s r; p <0.001).

### Changing of scores after intervention

On average, the students improved in all 28 items of the CCOG. The mean improvement across all items was 0.53 on a 5-point scale (Table 2), and progression was observed especially in following areas of communication: “encourages patient to discuss any additional points”, “establishes dates” and “closes interview by summarising briefly”. In contrast, other areas already had a high level in the first interviews and improved only slightly, e.g. “demonstrates respect” or “listens attentively”. Some areas such as “negotiates agenda” and “determines and acknowledges patients ideas” received more or less the same ratings for the first and second interviews.

**Table 2:** Mean scores of the CCOG items for the first and second interview *

**Item**	**Pre**	**Post**	**Difference**	
	**mean**	**mean**	**mean**	**(95% CI) **^**†**^
*Section: Initiating the Session*	*2.9*	*2.6*	*0.3*	*(0.1 - 0.5)*
Greets patient	2.0	1.8	0.2	(-0.1 - 0.5)
Introduces self and role	2.9	2.6	0.3	(-0.2 - 0.9)
Demonstrates respect	2.0	2.0	0.0	(-0.2 - 0.3)
Identifies and confirms problems list	3.1	2.5	0.6	(0.2 - 1.1)
Negotiates agenda	4.2	4.1	0.1	(-0.4 - 0.6)
*Section: Gathering Information*	*2.8*	*2.3*	*0.5*	*(0.2 - 0.9)*
Encourages patient to tell story	3.0	2.2	0.7	(0.3 - 1.2)
Appropriately moves from open to closed questions	3.1	2.4	0.7	(0.2 - 1.1)
Listens attentively	2.3	1.9	0.4	(0.1 - 0.8)
Facilitates patient’s responses verbally and non-verbally	2.6	2.1	0.5	(0.1 - 0.9)
Uses easily understood questions and comments	2.3	2.1	0.2	(-0.2 - 0.6)
Clarifies patient´s statements	2.8	2.5	0.3	(-0.1 - 0.7)
Establishes dates	3.5	2.5	1.0	(0.5 - 1.5)
*Section: Understanding Patient´s Perspective*	*3.4*	*2.8*	*0.5*	*(0.2 - 0.9)*
Determines and acknowledges patient´s ideas re: cause	3.7	3.4	0.3	(-0.2 - 0.9)
Explores patient´s concerns re: problem	3.1	2.5	0.6	(0.2 - 1.0)
Encourages expression of emotions	3.1	2.8	0.3	(-0.1 - 0.7)
Picks up/responds to verbal and non-verbal clues	3.4	2.8	0.6	(0.2 - 0.9)
*Section: Providing Structure to Consultation*	*3.3*	*2.9*	*0.4*	*(0.1 - 0.8)*
Summarises at end of a specific line of inquiry	3.9	3.4	0.5	(-0.1 - 1.0)
Progresses using transitional statements	3.3	2.9	0.4	(-0.1 - 0.9)
Structures logical sequence	3.3	2.8	0.5	(0.1 - 1.0)
Attends to timing	2.8	2.5	0.3	(-0.2 - 0.7)
*Section: Building Relationship*	*2.7*	*2.3*	*0.4*	*(0.1 - 0.8)*
Demonstrates appropriate non-verbal behaviour	2.6	2.2	0.5	(-0.1 - 0.9)
If reads or writes, doesn´t interfere with dialogue/rapport	2.7	2.3	0.4	(0.0 - 0.8)
Is not judgemental	2.4	2.1	0.3	(-0.1 - 0.8)
Empathises with and supports patient	2.9	2.4	0.5	(0.1 - 1.0)
Appears confident	3.1	2.5	0.6	(0.1 - 1.1)
*Section: Closing the Session*	*3.7*	*2.7*	*1.0*	*(0.6 - 1.4)*
Encourages patient to discuss any additional points	4.0	2.9	1.1	(0.6 - 1.6)
Closes interview by summarising briefly	3.9	3.0	0.9	(0.4 - 1.4)
Contracts with patient re next steps	2.9	2.0	0.9	(0.4 - 1.5)
Total score	3.1	2.6	0.5	(0.2 - 0.8)

* 5-point scale of all items; pre and post (scale: 1 = excellent, 5 = deficient).

† 95% confidence interval.

The more technically-orientated communication skills, as measured by six CCOG items, improved from 3.4 for the first interview to 2.6 in the second interview (difference: 0.8; 95%- confidence-interval: 0.5 to 1.1; p < 0.0001). The respective scores for the emotional items were 2.7 and 2.3 (difference: 0.4; 0.07 to 0.1; p = 0.023). The improvement for the technical items was, in some cases, twice as high as for the emotional ones. For example, the students’ ability to “close the session” improved from 3.7 to 2.7 (mean difference: 1.0; 95% CI 0.6-1.4) while the ability to “build a relationship” improved from 2.7 to 2.3 (0.4; 0.1-0.8).

We could not find any association between age, preparatory training or number of semesters and overall improvement or improvement in any areas of communication (data not shown), but we found some considerable gender differences.

### Gender differences

**Figure 2:**
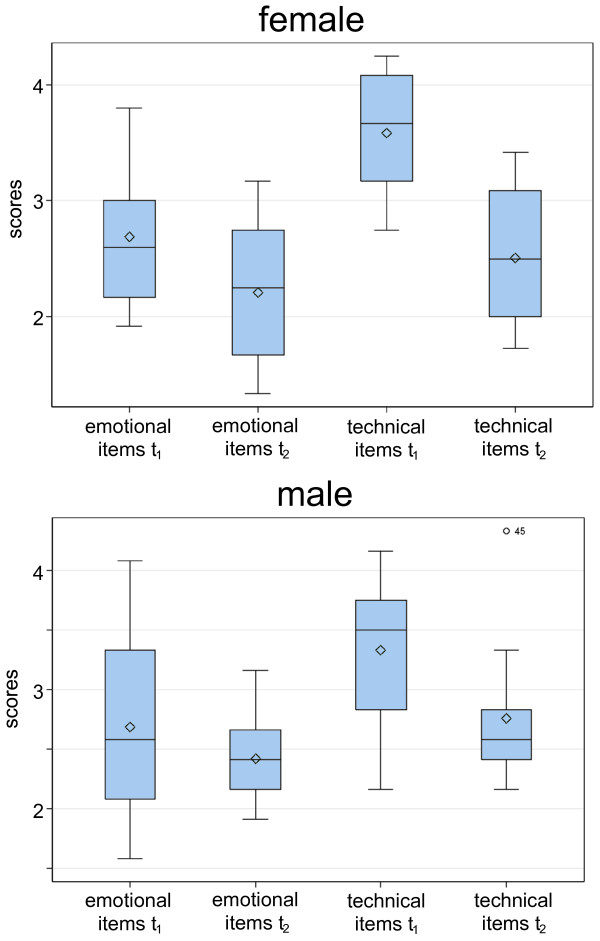
Changing of “technical” and “emotional” communication skills before and after the medical communication skills course.

The overall score for women improved from 3.2 (SD 0.4) for the first interview to 2.5 (SD 0.5) for the second interview (difference: 0.7; 95%-confidence-interval: 0.3 to 1.1; p = 0.0019); male students improved from 3.0 (SD 0.7) to 2.7 (SD 0.4; difference: 0.3; -0.14 to 0.8; n.s.). Female students especially improved their technical communication skills from 3.6 to 2.5 (difference: 1.1; 0.7 to 1.5; p < 0.0001), but their emotional communication skills only from 2.7 to 2.2 (difference: 0.5; 0.1 to 0.9; p = 0.044) (Figure 2). Female students improved most in their ability to encourage patients to discuss additional points (from 4.5 to 2.9; p < 0.0001). Another interesting gender difference is the degree of confidence (item "appears confident"). While males started with an average score of 2.9 and improved slightly to 2.6 (n.s.), their female peers started worse (3.2) but improved significantly to 2.3 (p=0.011).

### Interview length

The mean interview length was 5.6 minutes (SD: 1.6) in the first interview and this increased to 8.9 (3.1) minutes for the second interview (p < 0,0001). In both interviews, female students talked longer with SP than male students (first interview: 6.6 min. vs. 4.7 min.; second interview: 10.0 min. vs. 7.9 min.). Most importantly, while the length of the first interview correlated with the CCOG overall scores (Pearson’s r: 0.59; p < 0.0001), the length of the second interview did no longer correlate with the overall scores (0.06; n.s.). This was also true for the correlation of the change in interview length and change of the overall scores (Pearson’s r: 0.03; n.s.). That is to say, the raters did not give better scores merely because students talked longer. Female students had a bigger increase in consultation length and–independently–in the scores than their male peers.

## Discussion

Communication training in our basic clinical skills course significantly improved the communication skills of medical students. Especially the technical aspects of the medical interview were impressive and significant while the improvement in other areas was small and not significant. Female students benefited more from the training than male students. After training, the length of the interview significantly increased, but the duration of the interview did not correlate with the raters’ scores.

### Strength and weakness

The sample of students for this study was randomly selected so that they seem to be representative for third-year medical students. The random allocation of the consultation videos and the large number of raters who did not know the participating students may have contributed to the validity of the results.

In this small-scale study, it was not possible to include a control group or “waiting group” with cross-over design because of the curricular framework. This threatens the internal validity of our results—for several reasons [24]: Effects from uncontrolled studies are often greater than those from controlled studies. A test-retest effect may have happened so that the improvement in communication skills is not a result of our instruction but of repeated testing on the same activities. In this case, only the skills needed for this special task would have improved but not the broad range of procedural skills we have taught. Moreover, secular trends and other influential events which might have occurred during the intervention, could have affected the outcome. However, since we observed the effect of our intervention within a rather limited time frame such ‘history threats’ to the internal validity are rather unlikely. Since it is, on principle, impossible, to attribute the observed changes to our intervention on basis of this before-after design, conclusions should be interpreted with caution.

Inter-rater reliability was not optimal. It is possible that the instruction for raters was too short such that they had not become sufficiently acquainted with our rating instrument. However, the total scores of two raters─as we had calculated from the single items─strongly agreed with the correlation coefficients in the Wong et al study [25]. We know also from the literature, that global scores highly correlate with scores generated from checklists [26-28]. So we believe that raters had a valid impression of students communication skills over the 28 items in the synopsis [28]. Whenever we used the total score or sum scores for more than 1 item in our analysis, for example to compare male and female students or to analyse associations between the communication quality and the length of the interviews, our analysis seems valid.

The CCOG, designed in Canada and Cambridge in 1989, does perhaps not fully fit with our German teaching environment, but for comparability with international literature and after researching other possibilities, we choose this validated instrument as the best available [29].

### Which communication skills can be taught?

As Aspegren described [30], some communication skills such as “clear questions free of medical jargon” can be taught better than others like “initiating the session” or “stress a time frame for conversation”. Accordingly, we found that “technical skills” like “greets patient” and “encourages patient to discuss any additional points” scored significantly better after intervention. Such communication skills could obviously be trained easily and successfully.

Emotional skills such as “demonstrates respect” or “empathises with and supports patient”, did not change during intervention but scored highly from the outset. We were somewhat surprised about this result, because it cannot be taken for granted that young students already have a high level of these skills. Perhaps the good scores may have resulted from the difficulty of measuring empathy [31], so that raters gave high scores when they had difficulties to rate the students’ skills. As literature shows, empathy as such seems to diminish within medical education if it is not taught repeatedly [22,32,33]. To show this effect, our study would instead need to be conducted over a longer time-frame than the 12 weeks studied here. Of course, empathy is not reducible to a skill but a more complex issue or an attitude. Since it did not seem feasible to use an extra instrument measuring empathy, we relied on items within the CCOG. One of these (“empathises with and supports patient”) seems to cover empathy in an adequate way.

Other areas of communication did not change during intervention and remained insufficient: e.g. “negotiates agenda”, “attends to timing”. After reflecting on the CCOG and these items again, we recognised that they were either not taught during our course at all, or taught inconsistently between lecturers. The item with the lowest score (also before intervention) was “negotiates agenda”. This skill was definitely not mentioned in our script nor has it been a topic in our oral course lecture, expressing at the same time a high validity of the measurement.

### The gender bias

The difference between genders in medical communication is widely described. Here, we are in line with the literature: female students communicate in a more patient-centred, positive and emphatic manner. Accordingly, our female students could improve their ability to “empathise with and support patients” from 2.9 to 2.3, compared to male students (2.9 before and 2.9 after intervention) [34,35]. Despite interventions, this “invisible gender boundary” [34] remains over the years and can hardly be adjusted through medical education. However, the fact that our female students started with lower scores in some areas was surprising for us and we do not have an explanation. Perhaps the low level of self-assuredness which female students displayed before our intervention (see item: “appears confident”) also affected other areas of communication or influenced the raters’ perceptions of their overall competence.

### Length of interview

The length of the interview increased with all participants, but significantly more for female students. As Flocke et al. and Verdonk et al. [36] described in their cross-sectional observational studies [35], female doctors have longer consultation times and this was experienced as more “patient centred” by the patients themselves. Similarly to this report, the consultation time between male and female doctors in training in our study differed by about two minutes. Most importantly, communication scores and the degree of improvement from the first to the second interview did not correlate with changes in interview length. This is a nearly perfect finding for our study. While it is important that young students learn to spend time in doctor-patient interaction, the assessment of communication quality should not depend predominately on the consultation time itself. If this were the case, we would merely have to teach our students to spend more time with the patient and not to strengthen the broad range of their communication skills.

## Conclusion and Implications for further studies

Our “basic medical skills” course has established an innovative teaching model and measurably improved students’ communication skills, with female students benefiting in particular. Obviously, some “technical items” can be taught better than “emotional communication skills”, such as empathy or respect. Given the small sample size and a low inter-rater reliability, larger studies are necessary to confirm these results. Further studies are necessary to evaluate the effect of our course for individual students over a longer period of time. New aspects including students’ response to the teaching could be achieved by using qualitative methods like focus groups or interviews. In addition, although the CCOG provided a good base for this primary study, further studies of our course should adapt this instrument to specific educational objectives.

## Competing interests

The authors declare that they have no competing interests.

## Authors’ contributions

TF conceived the study. AS was responsible for the coordination of the project and the development of the manuscript. CW was responsible for data collection and made substantial contributions to the analysis and interpretation of data. CW and WH performed statistical analysis of the data. All authors have made contributions to the study design, acquisition and interpretation of data. All authors have been involved in drafting and revising the manuscript. All authors read and approved the final manuscript.

## Authors’ information

AS is a senior physician in the Department of General Practice and Family Medicine at the University of Göttingen who also works part-time as a board-certified general practitioner. She teaches communication skills and basic skills in general practice; the main focus of her research efforts lies in education research with a particular interest in measuring social and communication skills. TF is a board-certified general practitioner working full-time in a practice in Göttingen. He is also involved in teaching family medicine at the University of Göttingen. CW did her doctoral thesis in the Department of General Practice and Family Medicine and is currently working as a psychiatrist at the University of Göttingen Medical Centre. WH is a sociologist in the Department of General Practice and Family Medicine and his special interests are doctor-patient communication, patient illness narratives and health services research.
